# N-Acetyl-L-Cysteine Promotes Porcine Oocyte In Vitro Maturation and Subsequent Embryonic Development by Regulating Oxidative Stress

**DOI:** 10.3390/biology15171458

**Published:** 2026-08-26

**Authors:** Li Wang, Kelin Song, Qiuyu Meng, Feng Yang, Xuelei Han, Ruimin Qiao, Kejun Wang, Jun Bai, Tengfei Wang, Xiuling Li, Tong Yu, Xinjian Li

**Affiliations:** 1 College of Animal Science and Technology, Henan Agricultural University, Zhengzhou 450046, China; 2 Institute of Animal Science and Veterinary Medicine, Hainan Academy of Agricultural Sciences, Haikou 571100, China; 3 College of Animal Science and Technology, Hainan University, Haikou 570228, China

**Keywords:** NAC, porcine oocyte quality, relieved oxidative stress, mitochondrial function, IVF embryonic development

## Abstract

In vitro maturation (IVM) of oocytes is a critical initial step in mammalian in vitro production (IVP), and its quality directly influences the developmental competence of subsequent embryos. Oxidative stress is a major constraint on oocyte quality, which can be mitigated by exogenous antioxidants. In this study, porcine oocytes were matured in IVM medium supplemented with the antioxidant N-acetyl-L-cysteine (NAC; 0, 0.5, 1.5, and 4 mM) to evaluate its effects on maturation rate, antioxidant capacity, mitochondrial function, vitrification–warming survival rate, post-warming ROS levels, and early embryonic developmental rate. To elucidate the molecular mechanisms underlying the effects of NAC on porcine oocyte maturation, we conducted single-cell transcriptome sequencing. The results showed that, versus the control, 1.5 mM NAC substantially enhanced the IVM rate (*p* < 0.05), reduced ROS levels (*p* < 0.05), and increased mitochondrial activity, as assessed by MitoTracker, mitochondrial membrane potential (MMP), and ATP content (*p* < 0.05). These results suggest that 1.5 mM NAC relieves oxidative stress in oocytes and improves mitochondrial function. However, NAC addition showed no significant differences in vitrification–warming survival rate and post-warming ROS levels relative to the control group (*p* > 0.05). In addition, 1.5 mM NAC markedly improved the cleavage rate and blastocyst rate of porcine oocytes after in vitro fertilization (IVF, *p* < 0.05), and also enhanced the cleavage rate after parthenogenetic activation (PA, *p* < 0.05). Single-cell transcriptome sequencing revealed that, versus the control, differentially expressed genes (DEGs) identified after 1.5 mM NAC supplementation were mainly enriched in pathways related to oxidative phosphorylation (OXPHOS), spliceosome, and ubiquinone/terpenoid-quinone biosynthesis. Among these, the OXPHOS pathway showed the most significant enrichment, with upregulated expression of pathway-related genes such as *COX6C*, *CYCS*, and *SDHD*. The accuracy of the transcriptomic results was further validated by qPCR. In conclusion, supplementing with 1.5 mM NAC relieved oxidative stress, improved mitochondrial function, and thereby promoted oocyte maturation and improved oocyte quality, ultimately facilitating subsequent IVF early embryonic development.

## Simple Summary

The in vitro production of porcine embryos is important for animal breeding, reproductive research, and the preservation of valuable genetic resources. However, porcine oocytes are susceptible to damage from excessive reactive oxygen species (ROS) during in vitro maturation (IVM). This study evaluated whether N-acetyl-L-cysteine (NAC), a potent antioxidant, could protect porcine oocytes and improve their subsequent embryonic developmental competence. Among the concentrations tested, 1.5 mM NAC improved oocyte maturation, reduced oxidative damage, and enhanced the function of mitochondria, the cellular structures responsible for energy production. This treatment also increased the cleavage rate and blastocyst formation after fertilization and enhanced the cleavage rate after parthenogenetic activation (PA). However, this treatment did not significantly affect oocyte survival rate or ROS levels following vitrification–warming. Single-cell transcriptomic analysis revealed that the differentially expressed genes (DEGs) were mainly enriched in the oxidative phosphorylation (OXPHOS) pathway. These findings may help improve IVM methods for producing porcine embryos and support future advances in animal breeding and reproductive biotechnology.

## 1. Introduction

In vitro maturation (IVM) of oocytes is an essential step in embryo in vitro production (IVP), and the quality directly determines the success of subsequent embryonic development [1,2,3]. In recent years, although IVM systems for mammalian oocytes have been substantially optimized, the current efficiency of in vitro oocyte maturation remains lower than that achieved under in vivo conditions [4]. During oocyte IVM, oxygen tension [5], regulatory factors [6], temperature [7] and oxidative stress [8] can affect maturation quality. Among these factors, oxidative stress caused by the fundamental differences between the in vivo and in vitro environments is considered one of the major factors impairing oocyte quality [9,10]. In conventional IVM systems, incubators for in vitro culture are typically maintained at an oxygen tension of 20%, which is much higher than that in the oviductal and intrauterine environments (2–8%). This high oxygen concentration promotes electron leakage from the mitochondrial electron transport chain, resulting in excessive superoxide production [11]. In addition, temperature fluctuations and light exposure during oocyte retrieval, together with the intrinsic metabolism of oocytes and surrounding granulosa cells, can lead to ROS accumulation [7,12]. High intracellular ROS levels can cause cellular membrane damage, DNA double-strand breaks [13], and mitochondrial dysfunction [14], thereby activating apoptotic pathways and compromising oocyte quality and subsequent developmental potential. Numerous studies have demonstrated that antioxidant supplementation in oocyte maturation media can effectively alleviate oxidative stress during IVM [15,16].

The vitrification cryopreservation technique has been widely applied to the cryopreservation of oocytes from various mammalian species. However, the low-temperature treatment and exposure to high concentrations of cryoprotectants during this process not only cause direct damage to the oocyte’s cytoskeleton [17,18] and spindle structure [19] but also induce mitochondrial dysfunction [20] and oxidative stress [21,22,23], thereby impairing the developmental capacity of the oocyte. Studies have shown that supplementation with antioxidants, such as chlorogenic acid [24] and spermidine [25], during the oocyte vitrification process can effectively alleviate cryo-induced oxidative stress damage.

N-acetyl-L-cysteine (NAC) is a potent antioxidant and an acetylated derivative of cysteine. NAC contains a sulfhydryl group (-SH), which enables it to directly eliminate reactive oxygen species, including hydroxyl radicals and hydrogen peroxide, through chemical reactions. More importantly, after penetrating the cell membrane, NAC is hydrolyzed to L-cysteine, which subsequently combines with glutamate and glycine through condensation reactions to generate intracellular glutathione (GSH), the major antioxidant in cells [26]. The strong antioxidant activity of NAC has made it a common supplement in ovarian follicle and oocyte culture media. Adding 1.5 mM NAC to goat oocyte IVM medium enhanced oocyte quality and embryonic development by improving antioxidant capacity and metabolic activity [27]. Similarly, adding 1 mM NAC to sheep oocyte IVM medium produced equivalent beneficial outcomes [28]. In porcine oocyte IVM, addition of 1.5 mM NAC during either the 24–48 h or the entire 48 h maturation period significantly increased the number of oocytes with male pronuclei after IVF and reduced DNA fragmentation levels [29]. Addition of 1.5 mM NAC after 22 h of IVM also notably elevated the blastocyst rate of porcine oocytes [30]. In bovine oocytes, treatment with 1.0 mM NAC for 8 h during IVM significantly reduced intracellular ROS levels and increased GSH concentration [31]. Studies have further demonstrated that NAC can alleviate oxidative stress-induced damage in oocyte quality. During IVM, NAC supplementation effectively inhibited ZEA-induced decreases in porcine oocyte maturation rate and GSH content, as well as increases in ROS levels [32]. In mouse ovarian granulosa cells and follicular fluid under repeated COH-induced oxidative stress, NAC promoted Nrf2 nuclear translocation and upregulated SOD and GPx expression, thereby reducing ROS overload and improving oocyte quality [33]. NAC has also been reported to delay postovulatory oocyte aging [34]. Intraperitoneal injection of NAC in mice rescued follicular and oocyte developmental defects caused by *Kat8* deficiency [35]. During IVM of mouse oocytes, NAC treatment lessened the occurrence of chromosome segregation errors in *Sod1*-deficient oocytes [36]. In feeding experiments, dietary supplementation with NAC in conventional sow diets was shown to attenuate excessive activation of the ERK signaling pathway and improve uterine receptivity, thereby ameliorating adverse pregnancy outcomes caused by ovarian stimulation [37]. NAC administration after oocyte vitrification can improve the quality of vitrified-matured mouse oocytes [38].

Although previous studies have reported beneficial effects of NAC on the maturation rate and early embryonic development of in vitro cultured porcine oocytes, systematic experimental evaluations investigating NAC’s ability to alleviate oxidative stress and vitrification-induced damage in porcine oocytes, as well as its impact on subsequent embryonic developmental capacity, remain limited. Furthermore, the underlying molecular mechanisms of NAC action have not been fully elucidated. In this study, we systematically investigated the effects of adding different concentrations of NAC to porcine oocyte IVM culture medium on oocyte maturation, oxidative stress, mitochondrial function, vitrification-induced damage, and subsequent embryonic developmental capacity. Additionally, single-cell transcriptomic sequencing was performed to explore the molecular mechanisms underlying the effects of NAC on porcine oocyte IVM, providing new insights into strategies for improving porcine embryo IVP efficiency.

## 2. Materials and Methods

### 2.1. Main Reagents

Without specific indication, all reagents employed in this research were sourced from Sigma Aldrich (Burlington, MA, USA). The following reagents were used: TCM199 medium (11150-059, Gibco, Thermo Fisher Scientific Inc., Waltham, MA, USA), the following reagents were purchased from Beyotime Biotechnology (Shanghai, China): ROS assay kit (S0033S), mitochondrial red fluorescent probe MitoTracker Red CMXRos (C103), JC-1 kit (C2006), ATP assay kit (S0027) and Annexin V-FITC apoptosis detection kit (C10625), glutathione fluorescent probe Monochlorobimane (HY-101899, MedChemEXpress, Monmouth Junction, NJ, USA), dimethyl sulfoxide (67-68-5, Beijing Solarbio Science & Technology Co., Ltd., Beijing, China), Evo M-MLV reverse transcription premix kit (AG11728, Accurate Biotechnology (Human) Co., Ltd., Changsha, China), and SYBR Green Pro Taq HS premix qPCR kit (AG11701, AG, China).

### 2.2. Preparation of NAC Solution

A total of 1.6319 g NAC was weighed and dissolved in 20 mL double-distilled water to make a 500 mM stock solution, which was maintained at −80 °C. NAC was supplemented into the IVM medium at final concentrations of 0, 0.5, 1.5, and 4 mM. Prior to use, appropriate volumes of the NAC stock solution were added to the IVM medium to achieve the desired final NAC concentrations.

### 2.3. Oocyte Collection and IVM Culture

Porcine ovaries were freshly harvested from a local slaughterhouse in Zhengzhou. Ovaries were stored in a thermos flask with physiological saline pre-warmed to 37 °C and delivered to the laboratory within 2 h. After washing the ovaries, follicular fluid was aspirated from follicles measuring 3–6 mm using a sterile 10 mL syringe and transferred into Dulbecco’s phosphate-buffered saline (DPBS). Cumulus–oocyte complexes (COCs) with homogeneous cytoplasm—surrounded by at least three layers of cumulus cells—were selected and then placed in IVM medium supplemented with 0, 0.5, 1.5, and 4 mM NAC. Maturation was carried out at 38.5 °C in a humidified atmosphere of 5% CO_2_ for a total duration of 44 h. After maturation, the COCs were transferred into 0.5% (*w*/*v*) hyaluronidase solution and incubated at 38.5 °C for 3 min. The COCs were then gently pipetted repeatedly until the cumulus cells were completely removed. After being washed three times with IVM medium, the oocytes were examined under a phase-contrast microscope. Oocytes exhibiting the first polar body (PB1) were considered mature, and the maturation rates were calculated for the different experimental groups. The maturation rate was calculated as the ratio of the number of oocytes with PB1 extrusion to the total number of oocytes. The IVM culture medium was prepared using TCM-199 as the base solution, supplemented with 3.05 mM glucose, 0.91 mM sodium pyruvate, 0.57 mM cysteine, 10 ng/mL EGF, 10 ng/mL FSH, 0.5 µg/mL LH, 120 ng/mL IGF, 0.1% (*w*/*v*) polyvinyl alcohol, and 1% antibiotics and antibodies.

### 2.4. ROS and GSH Level Detection

Mature porcine oocytes were selected for ROS and GSH staining assays. The stock solution was prepared according to the instructions for the GSH staining kit. The ROS (1000×) and GSH (1000×) staining solutions were diluted with IVM medium to prepare the working solutions. Mature oocytes from each group were separately incubated with ROS or GSH working solution. Dark incubation steps were performed in a cell culture incubator: ROS for 20 min and GSH for 30 min. Subsequently, oocytes were washed at least three times with IVM medium to remove excess dye. Then, images were captured using an inverted fluorescence microscope. ROS fluorescence was detected in green, and GSH fluorescence was detected in blue. Finally, fluorescence intensity was analyzed using ImageJ 1.54 h software (National Institutes of Health, Bethesda, MD, USA).

### 2.5. Mitochondrial Activity, MMP and ATP Level Detection

The MitoTracker Red CMXRos fluorescent probe (1000×) and JC-1 staining solution (200×) were diluted with IVM medium to prepare the working solutions. JC-1 dye was fully dissolved by vigorous vortexing in the dark. Mature oocytes from different groups were then transferred into the corresponding staining working solutions and incubated in the dark for 30 min at 38.5 °C. After incubation, MitoTracker-stained oocytes were washed three times with IVM medium, whereas JC-1-stained oocytes were washed three times with prechilled 1× JC-1 washing buffer, with each wash lasting 5 min. Then, images were captured using an inverted fluorescence microscope. MitoTracker fluorescence was detected in red. JC-1 fluorescence was detected in red and green. Finally, fluorescence intensity was analyzed using ImageJ. Notably, the JC-1 fluorescence intensity ratio was calculated as the ratio of red to green fluorescence intensity.

Twenty mature oocytes from each group were picked and washed with PBS-PVA (0.1% PVA), then placed into ATP assay lysis buffer. After thorough vortexing, centrifugation was performed at 12,000× *g* and 4 °C for 5 min. The supernatant was collected and kept at 4 °C until further use. ATP standard curve solutions were prepared following the instructions of the ATP Assay Kit. After preparation, each well was preloaded with 100 µL ATP working solution and stood for 5 min, then 20 µL samples or standards were added sequentially. Chemiluminescence signals were then detected using a microplate reader. ATP content was calculated by substituting the measured luminescence values into a standard curve equation generated using Excel.

### 2.6. Annexin V Staining and Vitrification–Warming of MII-Stage Oocytes

Based on the Annexin V-FITC kit instructions, the working solution was prepared. Selected mature oocytes were placed in the working solution, then incubated at room temperature in the dark for 30 min. Afterwards, oocytes were washed three times with IVM medium for 5 min per wash. Then, images were captured using an inverted fluorescence microscope. Annexin V fluorescence was detected in green, and fluorescence intensity was quantified with ImageJ.

Selected mature oocytes from each group were subjected to vitrification. For vitrification, oocytes were first placed in equilibration solution at room temperature for 5 min, then transferred into vitrification solution for 30–35 s. Within 30 s, oocytes were quickly loaded onto a Cryotop and plunged into liquid nitrogen. The entire vitrification process was completed within 1 min, with up to 15 oocytes placed per Cryotop. For warming, Cryotop-stored oocytes were sequentially exposed to TCM199 medium containing 0.5 M sucrose and 20% FBS at 38.5 °C for 2 min, followed by stepwise dilution in TCM199 medium containing 20% FBS with sucrose concentrations of 0.5, 0.4, 0.2, and 0.1 M sucrose for 1 min each. The oocytes were then transferred to TCM199 medium containing 10% FBS for 3 min and recovered in IVM medium in a cell culture incubator for 2 h. After recovery, the survival rate was assessed, and ROS staining was performed (same as Section 2.4).

### 2.7. IVF, Parthenogenetic Activation (PA) and Embryo Culture

Oocytes matured in vitro for 44 h were gently pipetted to retain 2–3 layers of cumulus cells and were then transferred into mTBM for fertilization. Then, 1 mL of fresh boar semen was slowly added along the 15 mL tube wall into the PureSperm 40 and PureSperm 80 solutions that had been preheated at 38.5 °C for 30 min, and the mixture was centrifuged at 300× *g* for 10 min. Then, the lower sperm pellet was collected, 3 mL of HEPES solution that had been prewarmed at 38.5 °C for 12 h was added, and the mixture was centrifuged at 400× *g* for 5 min. The sperm pellet was collected and diluted with mTBM medium pre-equilibrated for 12 h in a cell culture incubator to achieve a final concentration of 5.0 × 10^6^ sperm/mL. After gentle mixing, 5 µL of the sperm was added to the mTBM droplet containing oocytes. At 6 h post-insemination, the oocytes were washed to remove residual sperm, transferred to PZM-3, and cultured at 38.5 °C with 5% CO_2_.

Mature oocytes were selected and incubated in ionomycin (ION; final concentration, 5 µM) for 4 min, followed by incubation in IVM medium for 5 min. The oocytes were then washed three times with PZM-3 and then placed in 6-dimethylaminopurine (6-DMAP; final concentration, 2 mM) for activation for 4 h. Finally, the oocytes were washed three times and cultured in PZM-3. All media were pre-warmed (ION, 6D, and PZM-3) for 2 h. The incubation, activation, and equilibration steps were all performed in a cell culture incubator. After IVF or PA, the cleavage rate was evaluated on day 2 and the blastocyst rate was evaluated on day 7, respectively. The cleavage rate was calculated as the number of cleaved embryos to the total number of oocytes, whereas the blastocyst formation rate was calculated as the number of blastocysts to the number of cleaved embryos.

### 2.8. RNA Extraction, Library Construction, and Sequencing

Mature porcine oocytes were selected from each group, washed three times with PBS-PVA, and then placed in cell lysis buffer provided by the BGI Genomics Co., Ltd. (Shenzhen, China). Samples were stored at −80 °C, and four replicates were collected per group. Sequencing was performed by the BGI company. The RNA concentration ranged from 29.27 to 39.81 ng/µL, and the total RNA amount ranged from 526.9 to 716.6 ng. The main peak of the amplification products was located at 800–2000 bp. Samples met the required quality standards and satisfied the requirements for library construction. Total RNA was used to enrich poly(A)-tailed mRNA with oligo(dT) magnetic beads, followed by fragmentation. First-strand cDNA was synthesized with N6 random primers, and second-strand synthesis generated dsDNA. After end repair, 5′ phosphorylation, 3′ A-tailing, and adapter ligation (T-overhang), products were PCR-amplified, heat-denatured to ssDNA, and circularized via bridge-primer-mediated circularization to construct single-stranded circular DNA libraries, which met the library construction requirements for single-cell full-length transcriptome sequencing. After passing quality control, the libraries were sequenced on the DNBSQE platform, with a total of 8 samples analyzed, each yielding an average output of 6.25 Gb of data. The average alignment rate to the genome for the samples was 97.76%, while the average alignment rate to the gene set was 63.47%; a total of 17,319 genes were successfully detected.

### 2.9. Single-Cell Transcriptomic Data Analysis

Transcriptome sequencing data were processed using SOAPnuke (v 1.5.6) to remove reads with adapter contamination, high proportions of unknown bases (N), and low quality, thereby obtaining clean reads; HISTA2 (v 2.1.0) was used to align to the Sus scrofa reference genome (GCF_000003025.6_Sscrofa11.1). Quantitative gene expression was expressed as FPKM values. Differentially expressed genes (DEGs) analysis was performed with DESeq2 (v 1.4.5), and DEGs were defined according to the criteria of |log_2_FC| ≥ 0.58 and Q value < 0.05. The identified DEGs were then analyzed by Gene Ontology (GO) and Kyoto Encyclopedia of Genes and Genomes (KEGG) enrichment analyses using the phyper function from R (v 4.4.1). The GO and KEGG enrichment outcomes were integrated with gene annotation information, and candidate genes were prioritized among the DEGs for qPCR verification. The gene primer sequences are listed in Table 1.

### 2.10. Real-Time PCR Validation of Differentially Expressed Genes

Selected mature oocytes were washed three times with PBS-PVA, and 20 MII stage oocytes were then added to each tube containing oocyte lysis solution for RNA extraction. Using the Evo M-MLV reverse transcription premix kit and the SYBR Green Pro Taq HS premix qPCR kit, RNA was reverse transcribed into cDNA and then PCR-amplified in a 10 µL reaction volume. Each qPCR experiment was performed with four biological replicates, and each biological replicate included three technical replicates. After amplification, melting curve analysis was carried out to confirm the specificity of the PCR products. *β-actin* was used as the reference gene. During data processing, the mean Ct value from the three technical replicates was calculated and used to represent the corresponding biological replicate, and then the relative expression levels of target genes were calculated using the 2^−ΔΔCt^ method.

### 2.11. Statistical Analysis

In this study, each treatment group within a single independent IVM batch, per culture droplet containing multiple oocytes, was considered one experimental unit, and each independent batch from different experiment dates was a biological replicate. All experiments were conducted with at least three replicates. All values are expressed as mean ± standard error of the mean (SEM). Statistical comparisons of percentage outcomes were based on the percentage values calculated from each independent biological replicate. Fluorescence images were acquired using an inverted fluorescence microscope with identical acquisition parameters (Light, Exposure, and Gain) repeated for each batch. The images were then imported into ImageJ, where a region of interest (ROI) was delineated along the edge of the oocyte zona pellucida. A uniform threshold parameter was applied to eliminate background interference, and fluorescence intensity was measured across the entire ROI in batch mode. GraphPad Prism (GraphPad Prism 8.3.0, GraphPad Software, San Diego, California USA) was used for both data processing and graph generation. Statistical comparisons among multiple groups were performed using one-way ANOVA, supplemented by Dunnett’s post hoc test. Comparisons between two groups were performed using the *t*-test. *, *p* < 0.05 was regarded as significant, **, *p* < 0.01 was considered highly significant, ns, *p* > 0.05 indicated no significant difference.

## 3. Results

### 3.1. Effects of NAC on the IVM Rate of Porcine Oocytes

**Figure 1:**
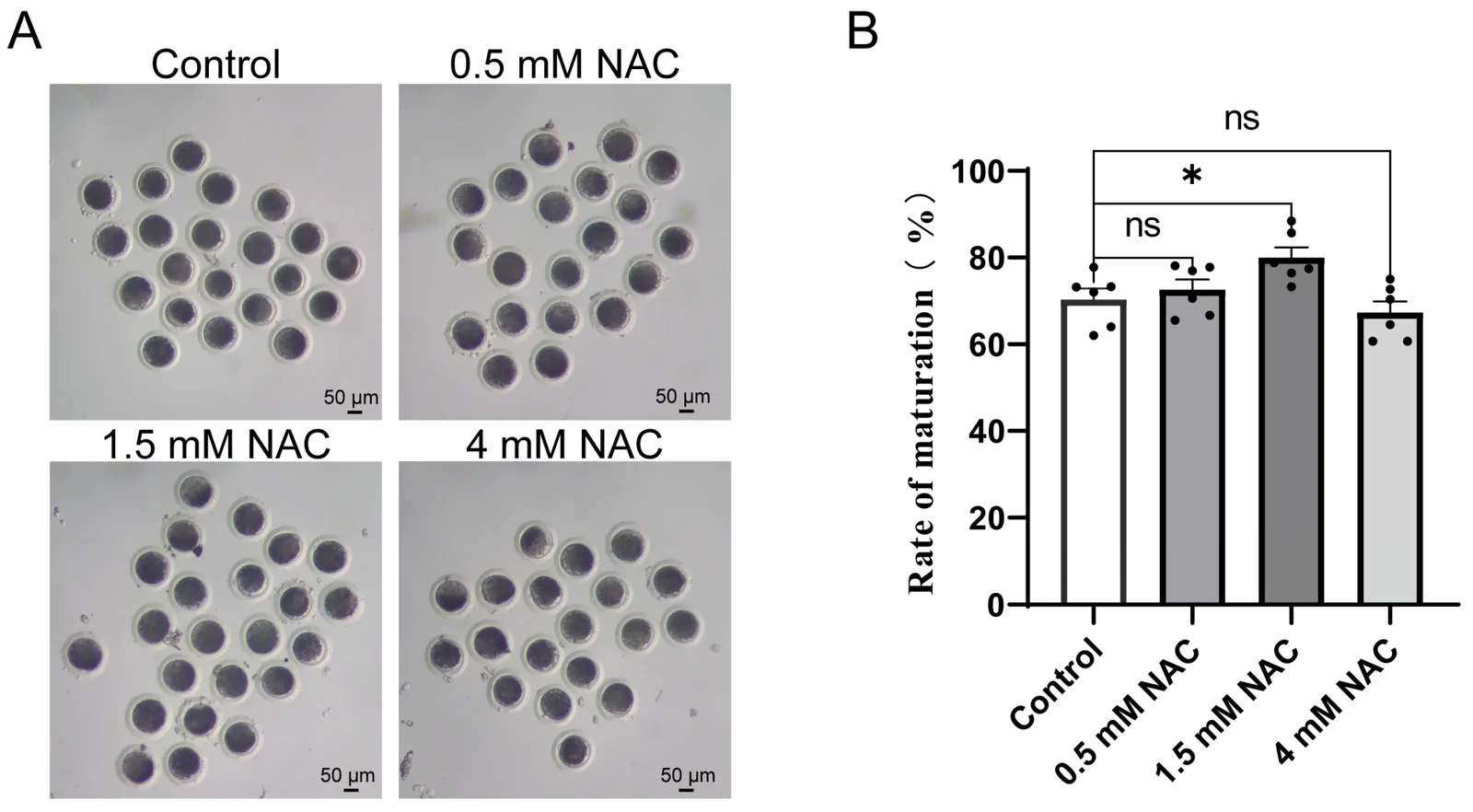
Effect of NAC supplementation on the maturation rate of porcine oocytes. Note: (**A**) representative images of PB1 extrusion after IVM in the various NAC-supplemented groups (Bar = 50 µm); (**B**) bar graph showing the oocyte maturation rates in the control and NAC-supplemented groups. Data are means ± SEM of six biological replicates. The “*n*” reported in Section 3.1 refers to the total oocyte count from six independent biological replicates. Each replicate included 32–35 oocytes. Data were analyzed using one-way ANOVA. *, *p* < 0.05, ns, *p* > 0.05.

In this experiment, we added various concentrations of NAC (0, 0.5, 1.5, and 4 mM) to the IVM medium and then measured the PB1 extrusion rate to determine its effect on oocyte maturation. Results showed that, versus the control (0 mM, 70.39% ± 2.47%, *n* = 206), treatment with 1.5 mM NAC notably improved the maturation rate (80.03 ± 2.73%, *n* = 204, *p* < 0.05). Supplementation with 0.5 mM (72.60 ± 2.35%, *n* = 212, *p* > 0.05) and 4 mM NAC (67.34 ± 2.54%, *n* = 193, *p* > 0.05) had no significant effect on the oocyte maturation rate (Figure 1A,B). Thus, 1.5 mM NAC was selected for subsequent experiments.

### 3.2. Effects of NAC Supplementation on ROS and GSH Levels in Porcine Oocytes

**Figure 2:**
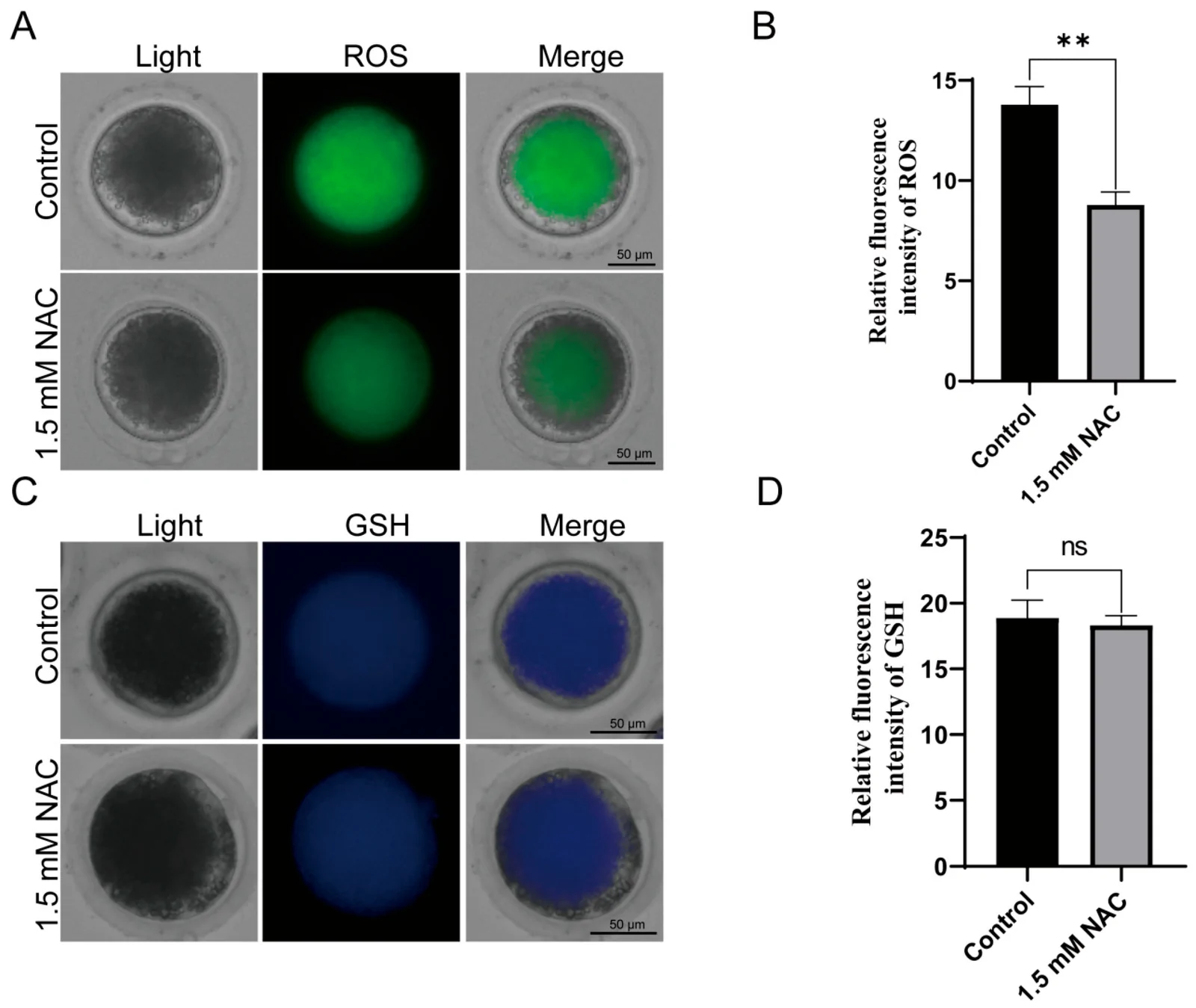
Effects of the addition of NAC on ROS and GSH levels in porcine oocytes. Note: (**A**) typical fluorescence images of ROS in various NAC-supplemented groups; (**B**) quantification of ROS fluorescence intensity in various NAC-supplemented groups; each biological replicate included 10–21 oocytes; (**C**) representative fluorescence images of intracellular GSH in various NAC-supplemented groups; (**D**) quantification of GSH fluorescence intensity in various NAC-supplemented groups; each biological replicate included 14–19 oocytes. Data are means ± SEM. Each experiment includes three independent biological replicates. The *t*-test was used to determine statistical significance. **, *p* < 0.01, ns, *p* > 0.05, bar = 50 µm.

ROS are key indicators for evaluating oocyte quality and subsequent early embryonic development. GSH content likewise serves as a key indicator of oocyte quality. The results indicated that, versus the control, 1.5 mM NAC markedly reduced ROS levels (Figure 2A,B, *p* < 0.05), but did not significantly affect the GSH content in porcine oocytes (Figure 2C,D, *p* > 0.05). These results indicated that 1.5 mM NAC treatment significantly reduced ROS levels in porcine oocytes and relieved oxidative stress.

### 3.3. Effects of NAC Addition on Porcine Oocyte Mitochondrial Function

**Figure 3:**
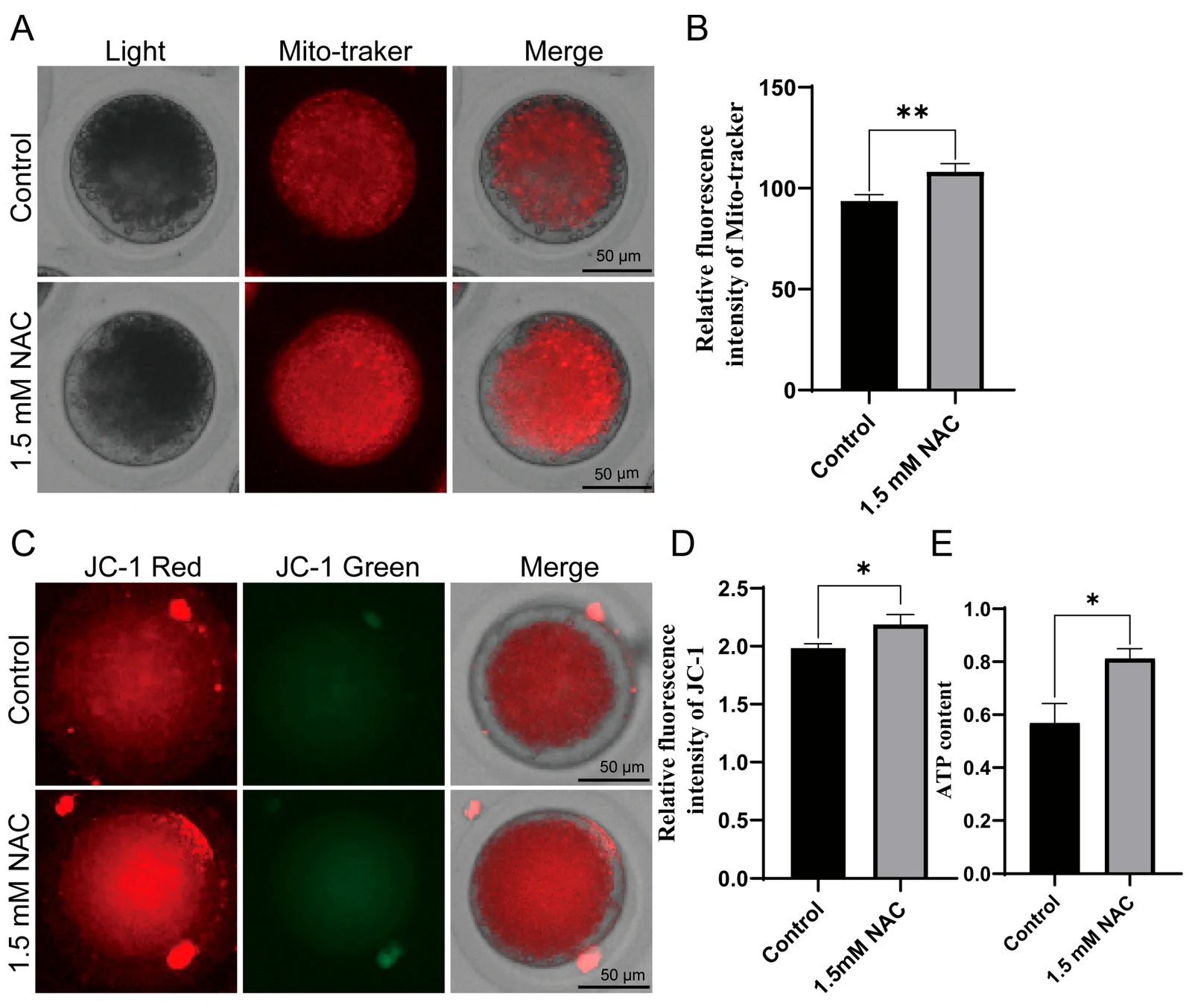
Effects of NAC addition on porcine oocyte mitochondrial function. Note: (**A**) typical fluorescence images for MitoTracker in various NAC-supplemented groups; (**B**) MitoTracker fluorescence intensity quantification in various NAC-supplemented groups, each biological replicate included 15–22 oocytes; (**C**) typical fluorescence images of JC-1 in various NAC-supplemented groups; (**D**) JC-1 fluorescence intensity quantification in various NAC-supplemented groups, each biological replicate included 12–16 oocytes; (**E**) ATP content quantification in various NAC-supplemented groups, each replicate included 20 oocytes. Data are means ± SEM. Each experiment includes three independent biological replicates. The *t*-test was used to determine statistical significance. *, *p* < 0.05, **, *p* < 0.01, bar = 50 µm.

Intact mitochondrial activity and a stable MMP are essential for driving oxidative phosphorylation (OXPHOS) and ATP generation, with ATP content reflecting the energetic reserves and metabolic state of oocytes. As shown in Figure 3, supplementation with 1.5 mM NAC notably elevated mitochondrial activity in oocytes (Figure 3A,B), MMP levels (Figure 3C,D), and ATP content (Figure 3E, *p* < 0.05). These results showed that 1.5 mM NAC could enhance mitochondrial activity, MMP, and ATP content, thereby improving mitochondrial function in porcine oocytes.

### 3.4. Effects of NAC on Early Apoptosis and Vitrification of Porcine Oocytes

**Figure 4:**
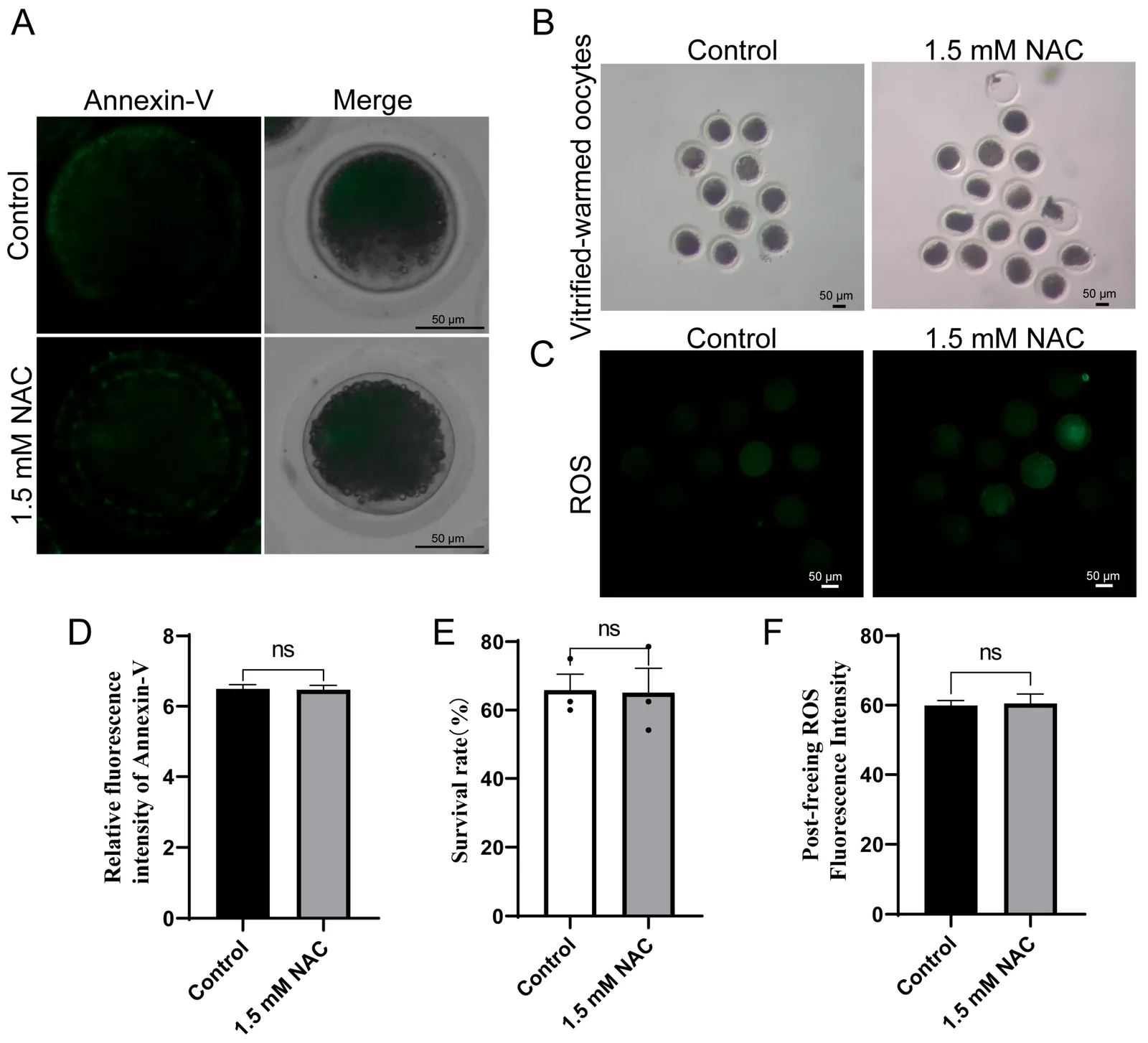
Effects of NAC supplementation on early apoptosis, post-warming survival rate, and ROS levels after vitrification in porcine oocytes. Note: (**A**) typical fluorescence images of Annexin-V in different NAC-supplemented groups; (**B**) typical micrographs of oocytes after vitrification–warming in different NAC-supplemented groups; (**C**) typical fluorescence images of ROS levels after vitrification–warming in different NAC-supplemented groups; (**D**) Annexin-V fluorescence intensity quantification in different NAC-supplemented groups, each biological replicate included 10–20 oocytes; (**E**) survival rate in different NAC-supplemented groups after vitrification–warming; (**F**) ROS fluorescence intensity quantification in different NAC-supplemented groups after vitrification–warming, each biological replicate included 9–13 oocytes. Data are means ± SEM. Each experiment includes three independent biological replicates. The “*n*” reported in Section 3.4 efers to the total oocyte count from three independent biological replicates, each replicate with 14–18 oocytes. Data were analyzed using a *t*-test; ns, *p* > 0.05; bar = 50 µm.

Apoptosis is involved in the quality control mechanism during oocyte development. According to the results, relative to the control group, the addition of 1.5 mM NAC had no significant effect on oocyte apoptosis (Figure 4A,D, *p* > 0.05). Oocyte cryopreservation can provide an abundant and stable source of high-quality oocytes, and ROS levels after cryopreservation are a key indicator for evaluating the quality of cryopreserved oocytes. Compared with the control group (65.83 ± 4.640%, *n* = 44), supplementation with 1.5 mM NAC (65.08 ± 7.161%, *n* = 54) had no significant effect on the survival rate (Figure 4B,E) or ROS levels (Figure 4C,F, *p* > 0.05) after vitrification–warming.

### 3.5. Effect of NAC on the Development of Pig IVF and PA Embryos

**Figure 5:**
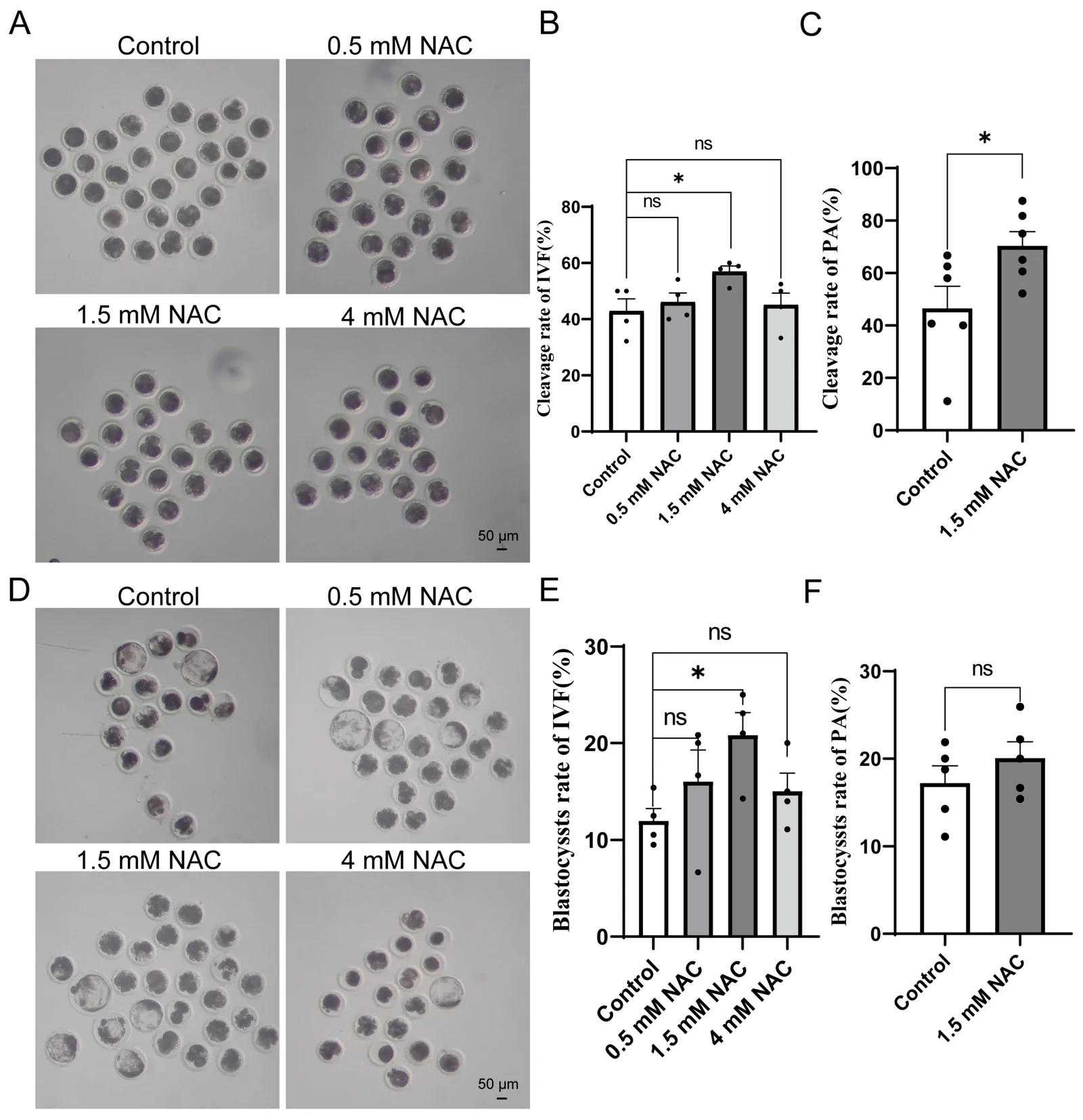
Effects of NAC supplementation on embryonic development of porcine oocytes after IVF and PA. Note: (**A**) typical micrographs of the cleavage rate on day 2 after IVF in different NAC-supplemented groups; (**B**) analysis of the cleavage rate after IVF in various NAC-supplemented groups, each biological replicate with 26–35 oocytes; (**C**) analysis of the cleavage rate after PA in the control and NAC-supplemented groups, each biological replicate with 26–28 oocytes; (**D**) typical micrographs of blastocysts on day 7 after IVF in different NAC-supplemented groups; (**E**) analysis of the blastocyst rate after IVF in various NAC-supplemented groups, each biological replicate with 14–20 oocytes; (**F**) analysis of the blastocyst rate after PA in the control and NAC-supplemented groups, each biological replicate with 18–23 oocytes. Data are means ± SEM of at least three biological replicates. The “*n*” reported in Section 3.5 refers to the total oocyte or embryo count from each independent biological replicate. IVF data were analyzed using one-way ANOVA. PA data were analyzed using a *t*-test. *, *p* < 0.05, ns, *p* > 0.05, bar = 50 µm.

IVF provides an artificial in vitro environment that facilitates sperm–oocyte fusion and supports the foundation for subsequent embryonic development. Parthenogenetic activation is an important technique in the study of reproductive processes, including gamete activation and fertilization. The results indicated that, versus the control, supplementation with 1.5 mM NAC significantly enhanced the cleavage rate (42.88 ± 4.37%, *n* = 141 vs. 56.97 ± 2.02%, *n* = 126, *p* < 0.05) and blastocyst rate (11.98 ± 1.29%, *n* = 69 vs. 20.84 ± 2.33%, *n* = 79, *p* < 0.05) of porcine oocytes after IVF. Supplementation with 0.5 mM (cleavage rate: 46.09 ± 3.30%, *n* = 122; blastocyst rate: 16.04 ± 3.25%, *n* = 62, *p* > 0.05) and 4 mM NAC (cleavage rate: 45.07 ± 4.26%, *n* = 102; blastocyst rate: 15.03 ± 1.85%, *n* = 53, *p* > 0.05) showed no significant difference in either the cleavage rate or blastocyst formation rate of porcine oocytes (Figure 5A,B,E). Supplementation with 1.5 mM NAC also significantly enhanced the cleavage rate (46.51 ± 8.42%, *n* = 161 vs. 70.35 ± 5.45%, *n* = 165, *p* < 0.05) after PA but had no significant effect on the blastocyst rate (17.21 ± 1.97%, *n* = 90 vs. 20.05 ± 1.90%, *n* = 112, *p* > 0.05, Figure 5C,D,F). These results indicated that supplementation with 1.5 mM NAC improved the embryonic developmental potential of IVM porcine oocytes.

### 3.6. Effects of 1.5 mM NAC on the Single-Cell Transcriptome of Pig Oocytes

**Figure 6:**
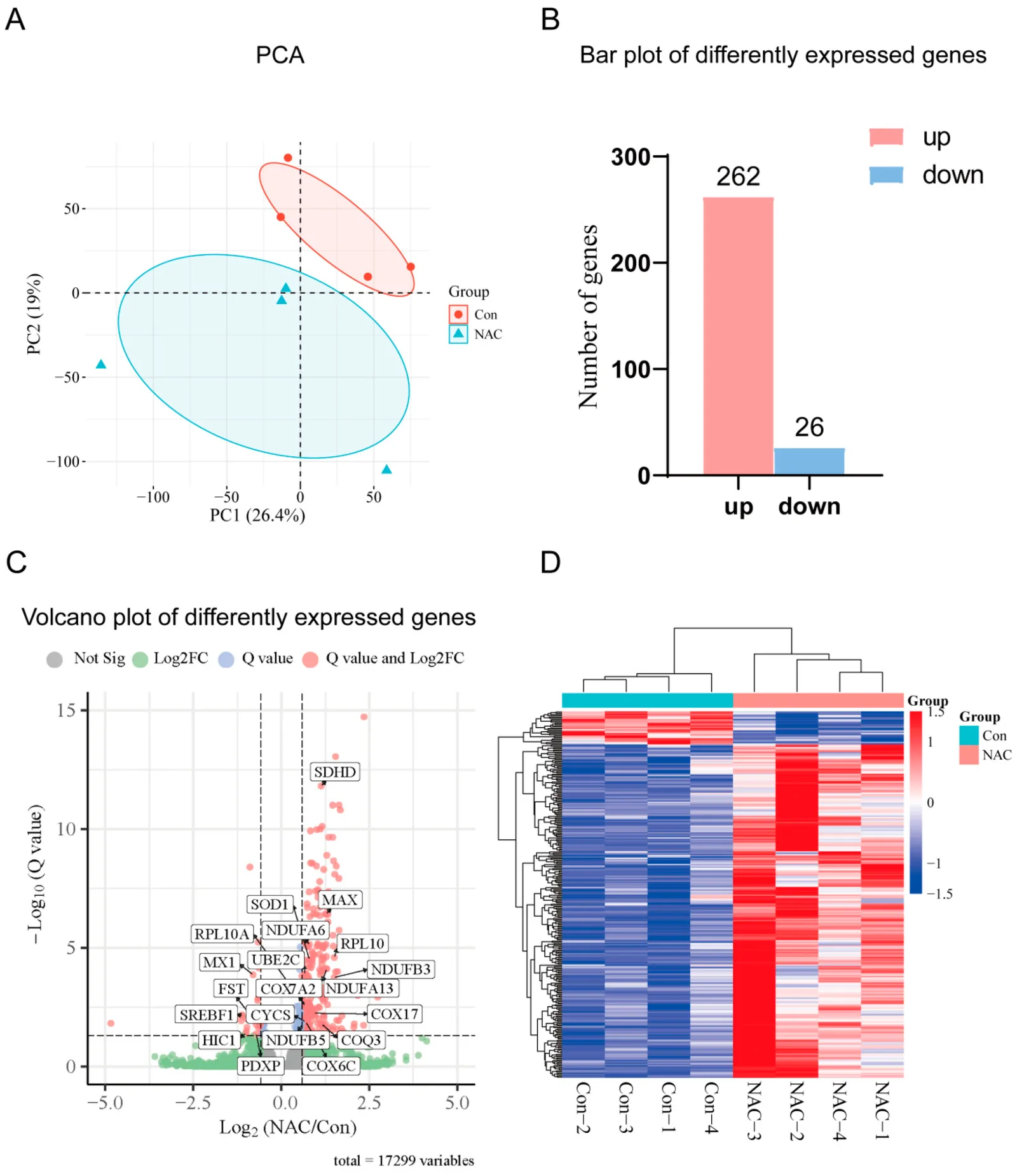
Gene expression analysis. Note: (**A**) PCA plot of the control and NAC-supplemented groups; (**B**) statistical analysis of the number of DEGs; (**C**) volcano plot of DEGs; and (**D**) clustering heatmap of DEGs.

Transcriptome sequencing analysis is a core approach for revealing the mechanism by which antioxidant supplementation acts on cells. In this study, principal component analysis (PCA) showed clear separation between the 1.5 mM NAC-supplemented and control groups, with PC1 and PC2 explaining 26.4% and 19% of the variance, respectively (Figure 6A). A total of 288 DEGs were identified, among which 262 were upregulated and 26 downregulated in the 1.5 mM NAC-supplemented group relative to controls (Figure 6B). The volcano plot highlighted several oocyte maturation-related genes, including *CYCS*, *COX6C*, *SDHD*, *SOD1*, and *RPL30* (Figure 6C). The clustering heatmap of DEGs demonstrated good biological reproducibility within each group and distinct transcriptional profiles between groups (Figure 6D). Table S1 presents the quality control of sequencing data. These results indicated that the transcriptome data were of sufficient quality for downstream pathway enrichment analysis.

**Figure 7:**
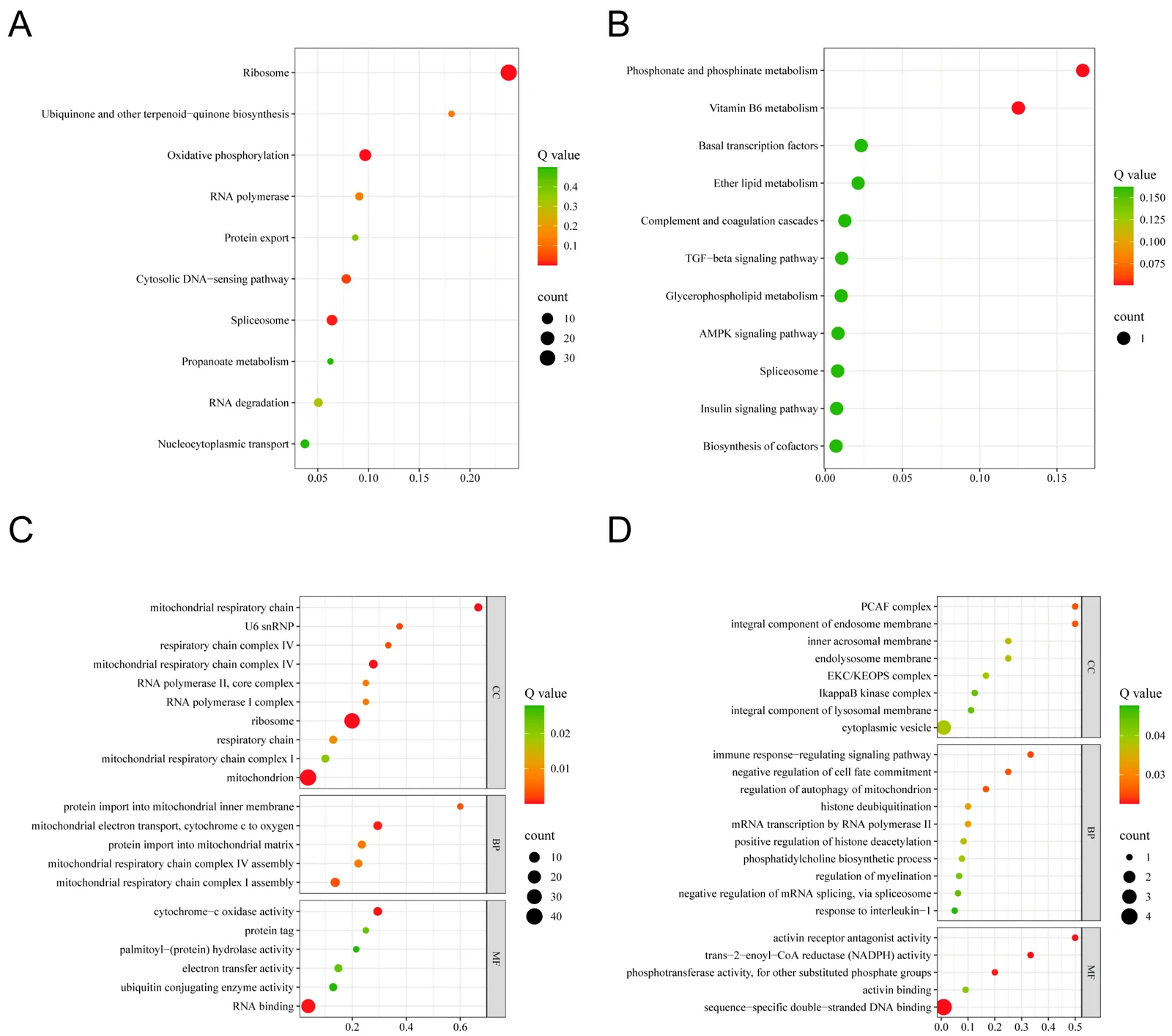
KEGG and GO enrichment scatter plots of differentially expressed genes. Note: (**A**,**B**) KEGG enrichment scatter plots of upregulated and downregulated DEGs; (**C**,**D**) GO enrichment scatter plots of upregulated and downregulated DEGs.

**Figure 8:**
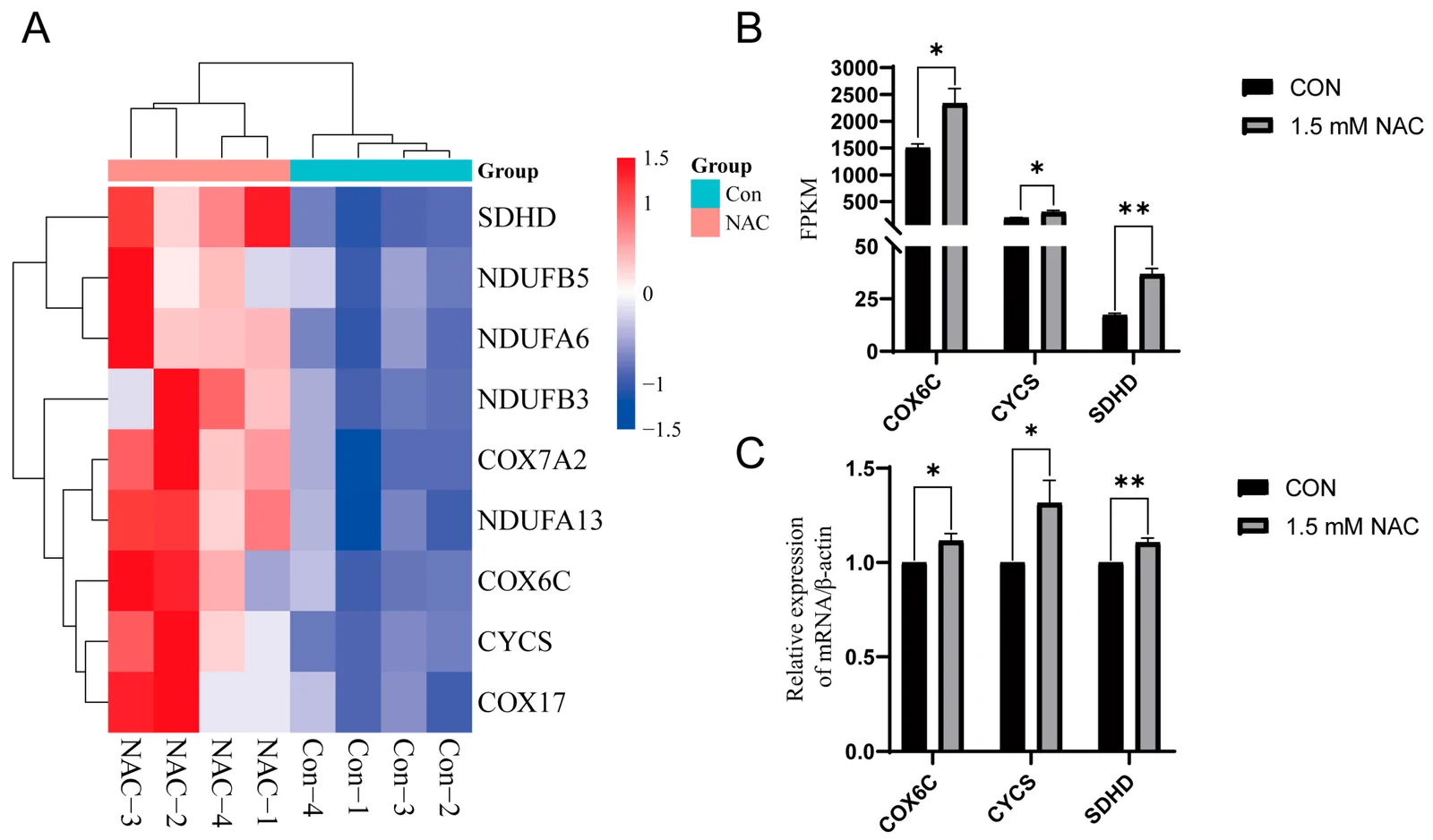
Expression patterns and qPCR validation of DEGs in the OXPHOS pathway. Note: (**A**) heatmap of DEGs in the OXPHOS pathway; (**B**) FPKM values of upregulated DEGs identified by transcriptome sequencing in the control and NAC-supplemented groups; (**C**) qPCR analysis of upregulated DEGs in the control and NAC-supplemented groups, each biological replicate with 20 oocytes. qPCR data are means ± SEM of four biological replicates. Data were analyzed using a *t*-test. *, *p* < 0.05, **, *p* < 0.01.

Next, KEGG and GO analyses were carried out on the DEGs. KEGG analysis showed that the upregulated DEGs were prominently enriched in OXPHOS, spliceosome, and ubiquinone/terpenoid-quinone biosynthesis, with OXPHOS being the most significantly enriched pathway (Figure 7A, *p* < 0.05), whereas downregulated DEGs mainly mapped to several metabolic pathways (Figure 7B). GO analysis revealed that upregulated DEGs were mainly assigned to mitochondrial respiratory chain complex I assembly, mitochondrial electron transport, cytochrome c to oxygen, and cytoplasmic translation (Figure 7C), while downregulated DEGs were associated with innate immune response (Figure 7D). The heatmap of DEGs in the OXPHOS pathway is presented in Figure 8A. Transcriptomic analysis revealed that *COX6C*, *CYCS*, and *SDHD* were upregulated in the 1.5 mM NAC-supplemented group compared with the control group (Figure 8B). These results were further validated by qPCR analysis, which confirmed the increased expression levels of these genes (Figure 8C), thereby supporting the reliability of the sequencing findings. Collectively, these results suggest that 1.5 mM NAC supplementation upregulates the OXPHOS pathway.

## 4. Discussion

The developmental competence of oocytes matured in vitro is considerably lower than that of their in vivo counterparts. Oxidative stress in oocytes, induced by fundamental discrepancies between the in vivo and in vitro environments, including temperature, light exposure, and oxygen tension, is considered a principal determinant of this phenomenon. The essence of oocyte oxidative stress lies in an imbalance between the intracellular oxidant production and antioxidant defense systems, characterized by excessive ROS production or insufficient antioxidant defense capacity, which subsequently causes cellular damage [39]. Excessive ROS accumulation disrupts oocyte integrity and meiotic progression, thereby affecting oocyte maturation quality and embryonic developmental potential. In the present study, supplementation with 1.5 mM NAC during IVM significantly increased the maturation rate of porcine oocytes versus the control, while reducing intracellular ROS levels. Previous studies have demonstrated that supplementation with NAC in the IVM medium can alleviate the adverse effects caused by oxidative stress and improve the oocyte maturation rate in animals such as cattle and sheep [27,31]. The findings of this experiment were consistent with previous studies.

GSH is the major antioxidant in cells and plays a role in scavenging ROS [40] and inhibiting mitochondrial dysfunction [41]. GSH deficiency can significantly increase ROS levels in oocytes, decrease MMP, and reduce lipid droplet reserves [42]. In the present study, there was no significant difference in GSH content and early apoptosis levels between the control and the 1.5 mM NAC groups.. This finding differed from previous reports on cattle and sheep, where NAC supplementation increased GSH levels. This discrepancy may be attributed to the ability of the free sulfhydryl group of NAC to directly react with ROS. This non-enzymatic reaction may occur rapidly and is independent of the GSH synthesis pathway, thereby alleviating oxidative stress in oocytes. However, NAC may also be consumed during this process. Therefore, although NAC serves as a precursor for GSH synthesis, its relatively low concentration may have limited the increase in GSH content. Nevertheless, this hypothesis requires further investigation. Future studies should assess the direct ROS-scavenging activity of NAC and the activities of enzymes involved in GSH metabolism to clarify the underlying mechanism.

Mitochondria are the primary sites of ATP. In the mitochondrial matrix, the tricarboxylic acid cycle generates electron carriers that deliver electrons to the mitochondrial electron transport chain located in the inner membrane. Meanwhile, respiratory chain complexes translocate protons from the matrix to the intermembrane space, generating MMP, driving OXPHOS, and yielding large amounts of ATP [43,44]. ATP levels are positively associated with oocyte developmental potential. Previous studies have shown that sheep oocytes with higher ATP levels exhibit improved fertilization and blastocyst rates [1,45]. However, electron leakage occurs in the mitochondrial electron transport chain, leading to ROS production [46]. Excessive ROS can damage the mitochondrial membrane, disrupt MMP, and subsequently reduce ATP synthesis [47]. Studies have shown that NAC can directly scavenge ROS and protect mitochondrial function [48]. In the present study, 1.5 mM NAC supplementation significantly increased mitochondrial activity, MMP, and ATP levels. These results indicated that NAC could improve mitochondrial membrane function and promote ATP synthesis, thereby enhancing the quality of porcine oocyte maturation in vitro.

Studies have found that in vitro-matured oocytes are more susceptible to oxidative stress due to excessive ROS accumulation, which can damage the cytoskeleton, increase the rate of abnormal fertilization, and impair pronuclear formation, ultimately compromising embryo quality [49]. In porcine oocyte IVM medium, supplementation with antioxidants such as urolithin A and resveratrol can improve the cleavage rate and embryo quality by alleviating oxidative stress-induced cellular damage [50,51]. In the present study, supplementation with 1.5 mM NAC notably elevated the cleavage rate and blastocyst rate of oocytes after IVF. However, Whitaker BD et al. reported that supplementation with 1.5 mM NAC did not affect the cleavage rate of IVF-derived porcine oocytes [30], which differs from the findings of the present experiment. We speculate that this difference may be due to differences in individual age, which could lead to differences in ovarian quality [52]. Whitaker BD et al. used ovaries from aged sows of at least 18 months old, whereas ovaries from young sows of 6–8 months were used in the present study.

The PA results indicated that the addition of 1.5 mM NAC significantly improved the cleavage rate of oocytes but had no significant effect on the blastocyst rate. This result differed from the IVF findings. Unlike IVF, PA bypasses fertilization-related events, such as sperm–egg fusion and paternal genomic contribution, and mainly reflects the cytoplasmic maturity and embryonic developmental potential of oocytes. The increased cleavage rates of both IVF and PA embryos following NAC supplementation further suggest that NAC improves oocyte maturation quality. However, NAC treatment did not markedly increase the PA blastocyst rate, suggesting that its beneficial effects on porcine oocytes primarily focus on regulating the fertilization process rather than comprehensively enhancing their basal metabolic potential. These differences may stem from distinct developmental regulatory mechanisms between IVF and PA embryos. Studies indicate that genomic activation in porcine embryos occurs at the 4-cell stage, a critical event during early embryogenesis that is influenced by both maternally expressed genes and sperm-derived miRNAs [53,54]. Consequently, IVF embryos may exhibit superior performance in genomic activation and subsequent development. Additionally, research shows that parthenogenetically developed embryos are more sensitive to environmental stressors [55]; thus, the failure to improve PA blastocyst rates may be attributed to heightened susceptibility to oxidative stress-induced damage coupled with the absence of paternal genetic input. However, this study only evaluated the cleavage rates and blastocyst rates in IVF and PA and did not statistically analyze indicators such as the number of inner cell masses or trophoblast cells to assess blastocyst quality. Therefore, the overall impact of NAC on embryo quality requires further validation.

Oocyte cryopreservation is an important component of assisted reproductive technologies and embryo bioengineering. Cryopreserved oocytes can provide an abundant source of gametes for technologies such as IVF, embryo transfer, and somatic cell nuclear transfer, without being limited by time or space [56]. During the vitrification–warming process, factors such as drastic osmotic and cryo-induced stress and ice crystal formation not only lead to a sharp increase in intracellular ROS levels but also cause various types of cellular damage, including mechanical damage, cytoskeletal disruption, abnormal spindle formation, and zona pellucida hardening [18,21,22,23,57,58]. In the present study, supplementation with 1.5 mM NAC during porcine oocyte IVM did not significantly affect post-vitrification survival rates or ROS levels. These findings are consistent with previous studies conducted in bovine oocytes, where the addition of 1 mM NAC during the IVF stage for 8 h failed to reduce ROS levels after vitrification–warming [59]. In contrast, NAC supplementation throughout the 44 h IVM period significantly reduced ROS levels in oocytes, indicating sustained antioxidant activity during maturation. We speculate that this discrepancy may arise from two potential factors. First, addition of NAC during IVM may allow sustained antioxidant activity, whereas the absence of NAC during vitrification–warming may limit its protective effects during this process. To validate this hypothesis, further experimental validation, such as adding NAC during the vitrification–warming process, is warranted. Second, NAC primarily targets oxidative stress-induced damage and cannot simultaneously counteract the multiple types of physical and chemical insults described above. It should be noted that our evaluation focused solely on post-vitrification–warming survival rates and ROS levels, without conducting a comprehensive assessment of mitochondrial function or spindle structure. Previous studies have indicated that NAC is most effective when added after vitrification to improve the quality of mature mouse oocytes but is not recommended for addition prior to vitrification [38]. Future research should investigate more comprehensive cryopreservation tolerance studies encompassing mitochondrial function, spindle morphology, and embryonic developmental capacity, while also exploring methods for direct NAC supplementation during the vitrification freezing process to better address the multifaceted damage associated with vitrification.

OXPHOS serves as the primary source of ATP production in oocytes and is essential for regulating the rate of oocyte maturation [60,61,62]. OXPHOS involves sequential electron transfer through the four multi-subunit complexes of the electron transport chain (ETC; complexes I–IV), which establishes a proton gradient across the inner mitochondrial membrane. This proton gradient then drives complex V, namely ATP synthase, to phosphorylate ADP into ATP, thereby providing energy required for cellular functions [63,64]. Studies have shown that, compared with normal oocytes, oocytes with maturation arrest or fertilization failure exhibit significantly decreased MMP and ATP content, whereas oocytes with higher OXPHOS activity show a significantly increased blastocyst rate [60,65,66]. Other studies have also shown that antioxidants, such as melatonin, improve oocyte developmental competence by regulating the OXPHOS pathway, and this effect has been confirmed in both human immature oocytes and porcine oocytes damaged by ochratoxin A [67,68]. Our data revealed that, after treatment with 1.5 mM NAC, the DEGs were notably enriched in the OXPHOS pathway, accompanied by significantly increased MMP and ATP levels and an improved blastocyst rate after IVF.

Succinate dehydrogenase subunit D (*SDHD*) is an essential component of mitochondrial electron transport chain complex II. It is responsible for catalyzing succinate oxidation and electron transfer, thereby contributing to ATP production required for energy-demanding processes such as oocyte maturation and spindle assembly [69]. Studies have shown that exposure to 25 μM juglone significantly downregulates *SDHD* expression in bovine oocytes and reduces oocyte quality by inhibiting the PI3K/AKT/mTOR pathway [70]. In contrast to the inhibitory effect of juglone, the present study showed that treatment with 1.5 mM NAC significantly upregulated *SDHD* expression in porcine oocytes, showing a clear contrast with the damaging effect of juglone. Cytochrome c (*CYCS*) functions as an electron carrier between complexes III and IV in the mitochondrial respiratory chain and is also a key molecule in the intrinsic apoptotic pathway [71,72]. By maintaining electron transport continuity and OXPHOS efficiency, CYCS contributes to mitochondrial energy metabolism and influences oocyte quality [71]. Cytochrome c oxidase subunit 6C (*COX6C*) is a component of cytochrome c oxidase, which is located in the mitochondrial inner membrane. This complex catalyzes electron transfer from reduced cytochrome c to oxygen and plays an important role in cellular OXPHOS [73,74,75]. Studies have shown that the *COX6C* subunit can participate in the assembly of subcomplexes, which may function as rate-limiting intermediates that regulate COX activity and OXPHOS [76]. It has also been reported that downregulation of *COX6C*, a gene related to the OXPHOS pathway, may be one of the reasons why the developmental competence of lamb oocytes is lower than that of adult sheep oocytes [77]. In the present study, treatment with 1.5 mM NAC markedly upregulated the expression of *COX6C*, *CYCS*, and *SDHD* in porcine oocytes, which indicates that upregulation of genes associated with the OXPHOS pathway is linked to NAC treatment, which may represent a potential mechanism by which NAC improves oocyte quality. Further functional studies using OXPHOS-specific inhibitors are required to confirm whether NAC enhances oocyte quality by activating the OXPHOS pathway.

## 5. Conclusions

In summary, the present study demonstrated that 1.5 mM NAC supplementation during porcine oocyte IVM reduced ROS accumulation, enhanced mitochondrial activity, MMP, and ATP levels, alleviated oxidative stress-related damage, and improved mitochondrial function, thereby improving porcine oocyte quality and subsequent early embryonic development after IVF. Transcriptomic analysis revealed that the OXPHOS pathway was significantly enriched in NAC-treated oocytes, and qPCR validation confirmed the upregulation of *SDHD*, *COX6C*, and *CYCS*. These results may indicate a potential link between NAC treatment and the activation of mitochondrial energy metabolism. However, whether NAC directly improves oocyte quality through OXPHOS pathway activation warrants further investigation via targeted functional assays. In addition to these findings, 1.5 mM NAC did not affect GSH content, early apoptotic status, or post-vitrification survival and ROS levels. Nevertheless, 1.5 mM NAC increased the cleavage rate after PA without improving blastocyst formation. Collectively, this study provides experimental insights for refining porcine oocyte IVM systems and establishes a foundation for future mechanistic exploration of NAC-mediated regulation of oocyte maturation.

## Figures and Tables

**Table 1:** List of differential gene primers.

Gene	Primer Sequence (5′→3′)	Annealing Temperature (°C)	Amplicon Size (bp)
*β-actin*	F: GACCACCTTCAACTCGATCAT	57.75	124
	R: GATCTCCTTCTGCATCCTGTC	57.89	
*COX6C*	F: GAACCCCGTCCATTTCTCCG	60.74	198
	R: GTTCAGCCACAGCAAACTTATAG	58.03	
*CYCS*	F: TGGTTAAACTTGAATGCGGAGTG	59.75	211
	R: GGCTCATGCCTTAACAGGCT	60.39	
*SDHD*	F: GCCTACTTCCAGCCGCTTAT	59.89	97
	R: ACTTGTCCAATGCCCCAGTG	60.54	

## Data Availability

All raw sequencing data generated in this study were deposited in NCBI and are accessible through accession number PRJNA1503661.

## Supplementary Materials

The following supporting information can be downloaded at: https://www.mdpi.com/article/10.3390/biology15171458/s1, Table S1: RNA quality assessment for single-cell sequencing libraries; Table S2: Clean reads filtering statistics for single-cell sequencing libraries; Table S3: Reference genome alignment statistics for single-cell sequencing libraries.
